# Association between arteriosclerosis index and lumbar bone mineral density in U.S adults: a cross-sectional study from the NHANES 2011–2018

**DOI:** 10.3389/fcvm.2024.1459062

**Published:** 2024-08-01

**Authors:** Chengxin Xie, Yu Ren, Qiang He, Chenglong Wang, Hua Luo

**Affiliations:** ^1^ Department of Orthopedics, Taizhou Hospital of Zhejiang Province Affiliated to Wenzhou Medical University, Taizhou, China; ^2^ Key Laboratory of Endocrine Glucose & Lipids Metabolism and Brain Aging, Ministry of Education, Department of Endocrinology, Shandong Provincial Hospital Affiliated to Shandong First Medical University, Jinan, China; ^3^ Department of Pharmacy, Taizhou Hospital of Zhejiang Province Affiliated to Wenzhou Medical University, Taizhou, China; ^4^ Department of Orthopedics, Shandong University of Traditional Chinese Medicine Affiliated Hospital, Jinan, China

**Keywords:** arteriosclerosis index, NHHR, lipid ratio, bone mineral density, adult, NHANES

## Abstract

**Background:**

The arteriosclerosis index, defined as the ratio of non-high density lipoprotein cholesterol to high density lipoprotein cholesterol (NHHR), has emerged as a novel biomarker for various diseases. The relationship between NHHR and lumbar bone mineral density (BMD) has not been previously examined.

**Methods:**

This cross-sectional study analyzed data from the National Health and Nutrition Examination Survey (NHANES) 2011–2018. NHHR was calculated as (total cholesterol—high-density lipoprotein cholesterol)/high-density lipoprotein cholesterol. Lumbar BMD was calculated to *Z* scores. Weighted multivariate linear regression, subgroup analysis, interaction analysis, generalized additive model, and two-piecewise linear regression were used.

**Results:**

A total of 8,602 participants were included. The negative association between NHHR and lumbar BMD was consistent and significant (Model 1: *β* = −0.039, 95% CI: −0.055, −0.023, *p* < 0.001; Model 2: *β* = −0.045, 95% CI: −0.062, −0.027, *p *< 0.001; Model 3: *β* = −0.042, 95% CI: −0.061, −0.023, *p* < 0.001). The linear relationship between NHHR and lumbar BMD was significantly influenced by body mass index (*p* for interaction = 0.012) and hypertension (*p* for interaction *= *0.047). Non-linear associations between NHHR and lumbar BMD *Z* scores were observed in specific populations, including U-shaped, reverse U-shaped, L-shaped, reverse L-shaped, and U-shaped relationships among menopausal females, underweight participants, those with impaired glucose tolerance, those with diabetes mellitus and those taking anti-hyperlipidemic drugs, respectively.

**Conclusions:**

NHHR exhibited a negative association with lumbar BMD, but varying across specific populations. These findings suggest that NHHR should be tailored to individual levels to mitigate bone loss through a personalized approach. Individuals at heightened risk of cardiovascular disease should focus on their bone health.

## 1 Introduction

Osteoporosis is the most common metabolic bone disorder, characterized by diminished bone mineral density (BMD) and deteriorated bone microarchitecture, resulting in increased bone fragility and fracture susceptibility (1). Fractures can occur in any bone, but hip and spinal fractures predominate, encompassing 42% of all osteoporotic fractures (2). Approximately 10.2 million individuals aged 50 and older in the U.S. are affected by osteoporosis, with over 40% of older adults exhibiting low bone mass, posing a heightened risk of progressing to osteoporosis (2). Reduced BMD is utilized as a diagnostic marker for osteoporosis, with the lumbar spine being a commonly measured site (3).

Dyslipidemia and osteoporosis are common diseases encountered globally. Emerging evidence suggests a positive correlation between cardiovascular diseases and osteoporosis, with lipid metabolism identified as a potential link (4, 5). Numerous studies have investigated the relationship between lipid profiles and BMD, yet the findings remain inconsistent and contentious (6–11). For instance, a cross-sectional study involving 10,039 U.S. adults revealed an inverse relationship between total cholesterol (TC) levels and BMD (6). Conversely, another study found a positive association between TC levels and lumbar BMD in Chinese males (7). The relationship between high-density lipoprotein cholesterol (HDL-C) and BMD also remains inconsistent. A meta-analysis study reported an elevated HDL-C levels were associated with osteoporosis (10), whereas another study reported a positive association between HDL-C and lumbar BMD (11). Current studies also failed to conclusively establish a clear relationship between low-density lipoprotein cholesterol (LDL-C) and BMD. These inconsistencies underscore the necessity of a comprehensive lipid index to determine its correlation with BMD accurately.

In recent years, the arteriosclerosis index has garnered increased attention as a superior biomarker compared to conventional lipid indicators for predicting cardiovascular risk (12–14). This index, calculated as the ratio of non-HDL-C to HDL-C (NHHR), reflects the balance between atherogenic and protective lipoproteins in the blood. It integrates all atherogenic cholesterol components, including LDL-C, very LDL-C, intermediate-density lipoprotein cholesterol, and lipoprotein (a), with HDL-C, which functions as an anti-atherogenic factor. The heterogeneity among various forms of non-HDL cholesterol might influence its association with BMD. In addition, other lipoprotein ratios, such as apolipoprotein B/apolipoprotein A1, are not typically included in standard testing and exhibit limited predictive performance (15). As a novel lipid indicator, NHHR accounts for the dual influence of non-HDL-C and HDL-C overcoming the limitations of single-lipid indicators. Encouragingly, NHHR has proven valuable for predicting various disease, including metabolic syndrome (15), diabetes (16), chronic kidney disease (17), kidney stones (18), nonalcoholic fatty liver disease (19), periodontitis (20), and depression (21). However, the relationship between the NHHR and bone health has yet to be investigated. Herein, this study explores this relationship in adults through a cross-sectional analysis, aiming to identify a clinically accessible indicator for BMD evaluation.

## 2 Methods

### 2.1 Data source

The National Health and Nutrition Examination Survey (NHANES) captures nationally representative statistics of the U.S. non-institutionalized civilian population biennially, employing a complex survey design and population-specific sample weights. Briefly, a series of household interviews were conducted, along with standardized physical examinations and laboratory tests in designated mobile examination centers (MEC) arranged across the country. The NHANES protocol was approved by the Ethics Review Committee of the National Center for Health Statistics, with all participants providing written informed consent. Detailed information can be accessed from the NHANES website (https://www.cdc.gov/nchs/nhanes/).

### 2.2 Population selection

**Figure 1:**
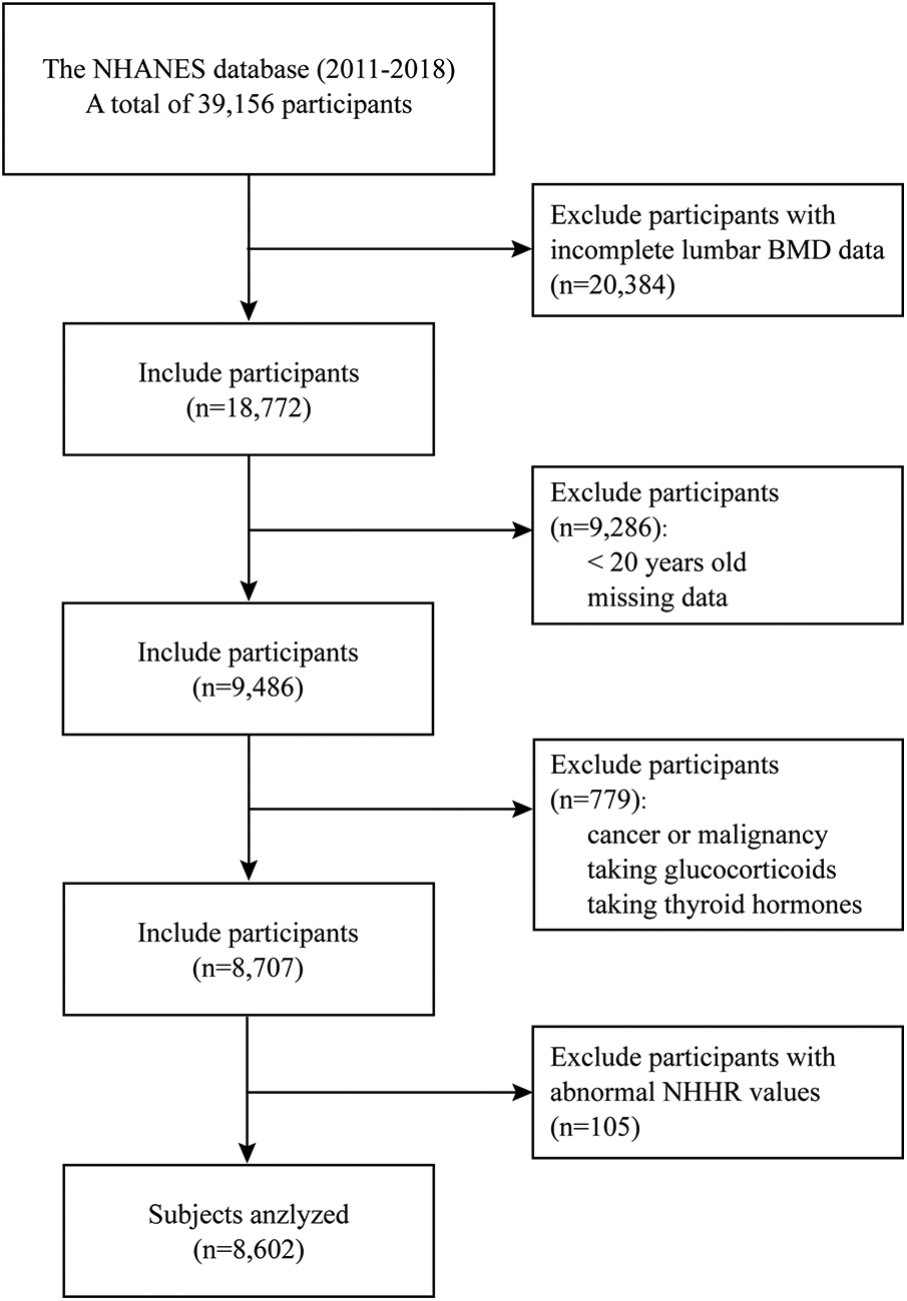
Flow chart of participants selection from the NHANES 2011–2018.

The population data were sourced from the NHANES database across four consecutive cycles (2011–2012, 2013–2014, 2015–2016, and 2017–2018), compassing a total of 39,156 participants (11). Initially, 20,384 individuals with incomplete lumbar BMD data were excluded. The dual-energy x-ray absorptiometry (DXA) examination was limited to participants aged 8 to 59 years, and pregnant females were ineligible. Individuals under 20 years old and those with missing data in NHHR and covariate variables were further removed. Participants with cancer or malignancy, taking glucocorticoids, and thyroid hormones were excluded. Finally, individuals with abnormal NHHR values [exceeding three times standard deviation (SD)] were excluded, leaving 8,707 participants (weighted *n* = 207,456,466) for analysis (Figure 1).

### 2.3 Exposure variable and outcome variables

The exposure variable was the NHHR, calculated as (TC–HDL-C)/HDL-C (12). TC and HDL-C were measured from the fasting serum samples. Participants were stratified based on the NHHR quartiles, including group Q1 (0.36,1.90), group Q2 (1.91, 2.65), group Q3 (2.66, 3.64), and group Q4 (3.65, 7.53).

The outcome variable was the lumbar spine BMD Z scores, representing the number of SDs by which an individual's BMD differs from the expected mean for the same age, gender, and race group (22). BMD values were evaluated via DXA during participants' visits to the MEC. The mean BMD for the scanned anteroposterior length from L1 to L4 was calculated and utilized for lumbar spine BMD reporting.

### 2.4 Covariates

Demographic data and variables potentially affecting NHHR and BMD were included as covariates: age, sex, race, education level, marital status, income, body mass index (BMI), waist circumference, smoking status, alcohol intake, hypertension, type 2 diabetes mellitus (DM) status, vigorous/moderate work activity (V/MWA), anti-hyperlipidemic drugs usage, alanine aminotransferase (ALT), aspartate aminotransferase (AST), alkaline phosphatase (ALP), albumin, serum calcium, serum phosphorus, serum 25-hydroxyvitamin D2 + D3 (25OHD2 + D3), and triglyceride (TG).

Age groups were stratified using a threshold of 35 years, given that bone mass gradually increases during early life, reaching its peak around ages 20 to 35 years (23). BMI was classified as underweight (< 18.5 kg/m^2^), normal weight (18.5 kg/m^2^ ≤ BMI <25 kg/m^2^), overweight (25 kg/m^2 ^≤ BMI <30 kg/m^2^) and obese (≥30 kg/m^2^) (24). Waist circumference was stratified into two groups based on the definition of central obesity (25): > 102 cm for males or >88 cm for females. Smoking status was divided into never (smoked less than 100 cigarettes in life), former (smoking >100 cigarettes in life but does not smoke now), and now (smoking ≥1 cigarette every day). V/MWA was defined as having done at least 10 min of V/MWA in a typical week. Hypertension was defined as systolic blood pressure ≥ 140 mmHg and/or diastolic blood pressure ≥ 90 mmHg, or self-reported hypertension along with the use of anti-hypertensive medication (26). DM status was classified into categories of no, impaired fasting glucose/glycemia (IFG), impaired glucose tolerance (IGT), and DM according to the standards set by the American Diabetes Association (27).

### 2.5 Statistical analysis

Appropriate weighting methodologies were employed to accommodate the complex sampling design in accordance with NHANES guidelines (28). Initially, the NHHR was divided into quartiles, with the lowest quartile (Q1) designated as the reference group. The basic characteristics of categorical variables were expressed as percentages (%) and continuous variables were described by means and standard error. Chi-square tests were employed to examine disparities among categorical variables, and analysis of variance (ANOVA) was utilized to analyze differences among continuous variables.

The association between the NHHR and lumbar BMD *Z* scores was investigated using a multiple linear regression model. According to the STROBE statement (29), Model 1 was unadjusted, Model 2 was minimally adjusted (adjusted for age, sex, and race), and Model 3 was fully adjusted (adjust for age, sex, race, education level, marital status, income, BMI, waist circumference, smoking status, hypertension, DM status, V/MWA, anti-hyperlipidemic drugs usage, alcohol intake, ALT, AST, ALP, albumin, serum calcium, serum phosphorus, serum 25OHD2 + D3, and TG). Based on Model 3, a multivariate linear regression model was applied to perform subgroup analyses of the linear association between NHHR and lumbar BMD *Z* scores across different subgroups. Interaction testing was performed to explore the potential effects of covariates on the association between NHHR and lumbar BMD *Z* scores.

The potential nonlinear association between NHHR and lumbar BMD *Z* scores was identified by generalized additive model (GAM) based on smooth curve fitting. When non-linearity was detected, a recursive algorithm was used to determine the significant inflection points, and a threshold effect analysis was conducted to assess the difference between the standard linear regression model and the segmented linear regression model.

All statistical analysis was performed using R software (version 4.3.3, http://www.R-project.org) and EmpowerStats (version 2.0, www.empowerstats.com). Statistical significance was defined as *p *< 0.05.

## 3 Results

### 3.1 Baseline characteristics of the participants

**Figure 2:**
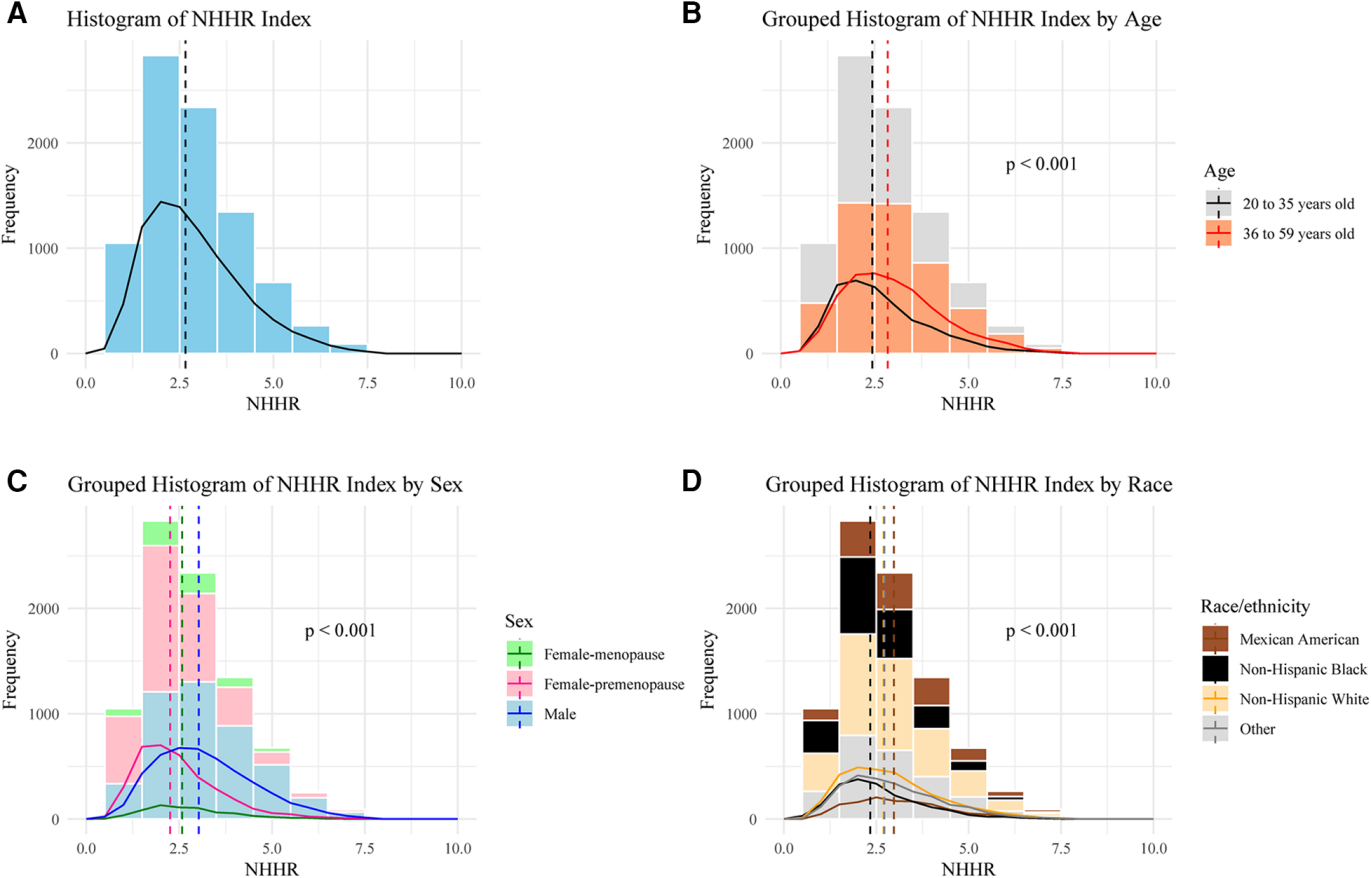
Frequency distribution of participants based on NHHR. (**A**) Overall, (**B**) Stratified by age, (**C**) Stratified by sex, (**D**) Stratified by race/ethnicity. One-way ANOVA was used to evaluate whether there are significant differences in NHHR values among different groups.

A total of 8,602 individuals were analyzed, comprising 4,517 male, 3,431 premenopausal female, and 654 menopausal female. The distribution of participants based on NHHR values is visualized in Figure 2. The median NHHR was higher among the males, Mexican Americans, and those aged over 35 years. The baseline characteristics of the included participants according to NHHR quartile are shown in Table 1. Compared to participants in the lower NHHR group, those in the NHHR Q4 group showed significantly lower lumbar BMD values and its *Z* scores (*p* < 0.001).

**Table 1:** Characteristics of the study population from NHANES 2011–2018.

Variable	Total	NHHR quartile				*p*-value
		Q1 (0.36–1.90)	Q2 (1.91–2.65)	Q3 (2.66–3.64)	Q4 (3.65–7.53)	
Age (years)	38.688 (0.252)	36.280 (0.456)	37.752 (0.365)	39.985 (0.383)	40.784 (0.368)	<0.001
Age-stratified (%)						<0.001
20–35 years old	43.485	52.962	47.800	37.881	35.132	
36–59 years old	56.515	47.038	52.200	62.119	64.868	
Sex (%)						<0.001
Male	54.039	34.837	47.712	59.513	74.861	
Female-premenopause	38.026	56.483	42.941	32.725	19.265	
Female-menopause	7.934	8.681	9.348	7.762	5.874	
Race/ethnicity (%)						<0.001
Non-Hispanic white	61.729	63.225	60.454	63.365	59.690	
Non-Hispanic black	11.505	14.670	13.316	9.692	8.267	
Mexican American	10.322	7.620	9.415	10.940	13.435	
Other	16.445	14.484	16.816	16.003	18.608	
Married (%)						< 0.001
No	49.243	56.892	50.556	45.625	43.762	
Yes	50.757	43.108	49.444	54.375	56.238	
Education (%)						<0.001
<High school	12.328	8.919	11.131	13.081	16.341	
High school	21.881	18.584	20.866	23.577	24.561	
>High school	65.790	72.498	68.003	63.342	59.098	
Smoke (%)						<0.001
Never	60.090	65.188	62.347	60.337	52.119	
Former	19.119	15.955	17.064	21.186	22.331	
Now	20.790	18.857	20.589	18.477	25.550	
Hypertension (%)						<0.001
No	73.733	83.011	76.486	70.620	64.499	
Yes	26.267	16.989	23.514	29.380	35.501	
DM (%)						<0.001
No	84.899	91.620	86.861	83.109	77.734	
IFG	4.474	2.146	4.185	4.651	7.033	
IGT	2.388	1.736	2.036	2.779	3.015	
DM	8.239	4.497	6.918	9.461	12.219	
V/MWA (%)						0.003
No	49.545	53.349	50.576	50.176	43.784	
Yes	50.455	46.651	49.424	49.824	56.216	
Anti-hyperlipidemic drugs (%)						0.21
No	56.718	58.233	54.874	55.293	58.579	
Yes	7.836	6.513	8.950	8.650	7.194	
Other	35.445	35.254	36.176	36.057	34.226	
Income to poverty ratio	2.945 (0.052)	3.018 (0.069)	2.980 (0.071)	2.979 (0.058)	2.795 (0.069)	0.019
BMI (kg/m^2^)	28.849 (0.143)	25.612 (0.173)	28.466 (0.185)	30.040 (0.202)	31.360 (0.213)	<0.001
Waistline (cm)	97.988 (0.353)	88.527 (0.417)	96.305 (0.433)	101.450 (0.478)	105.931 (0.512)	<0.001
ALT (U/L)	26.144 (0.254)	21.790 (0.521)	23.433 (0.324)	26.572 (0.417)	33.070 (0.637)	<0.001
AST (U/L)	25.041 (0.187)	24.567 (0.419)	23.951 (0.390)	24.899 (0.318)	26.823 (0.465)	<0.001
ALP (U/L)	66.665 (0.439)	61.887 (0.702)	64.408 (0.607)	68.556 (0.689)	71.974 (0.692)	<0.001
Serum phosphorus (mg/dl)	3.706 (0.010)	3.744 (0.019)	3.722 (0.017)	3.676 (0.017)	3.681 (0.019)	0.036
Serum calcium (mg/dl)	9.375 (0.007)	9.356 (0.011)	9.362 (0.010)	9.374 (0.011)	9.410 (0.010)	<0.001
Albumin (g/dl)	4.328 (0.007)	4.331 (0.011)	4.314 (0.012)	4.323 (0.010)	4.345 (0.010)	0.085
TG (mg/dl)	143.108 (1.902)	79.490 (0.749)	107.630 (1.334)	149.898 (2.118)	239.432 (3.945)	<0.001
TC (mg/dl)	189.928 (0.731)	167.498 (1.054)	179.286 (0.951)	195.128 (1.216)	218.907 (1.209)	<0.001
HDL-C (mg/dl)	52.774 (0.330)	68.902 (0.525)	54.938 (0.294)	47.675 (0.298)	39.089 (0.254)	<0.001
Alcohol intake (g)	14.494 (0.650)	18.855 (1.030)	13.906 (0.895)	12.357 (1.108)	12.830 (1.218)	<0.001
Serum 25OHD2 + D3 (nmol/L)	65.931 (0.790)	68.771 (1.151)	66.760 (1.030)	66.315 (1.073)	61.662 (0.885)	<0.001
Lumbar spine BMD (g/cm^2^)	1.039 (0.002)	1.059 (0.004)	1.045 (0.004)	1.033 (0.005)	1.018 (0.004)	<0.001
Lumbar spine BMD *Z* scores	0.025 (0.017)	0.094 (0.032)	0.045 (0.030)	0.012 (0.029)	−0.054 (0.031)	<0.001

Categorical variables were expressed as percentages (%).

Continuous variables were described by means and standard error.

### 3.2 Association between NHHR and lumbar BMD

**Figure 3:**
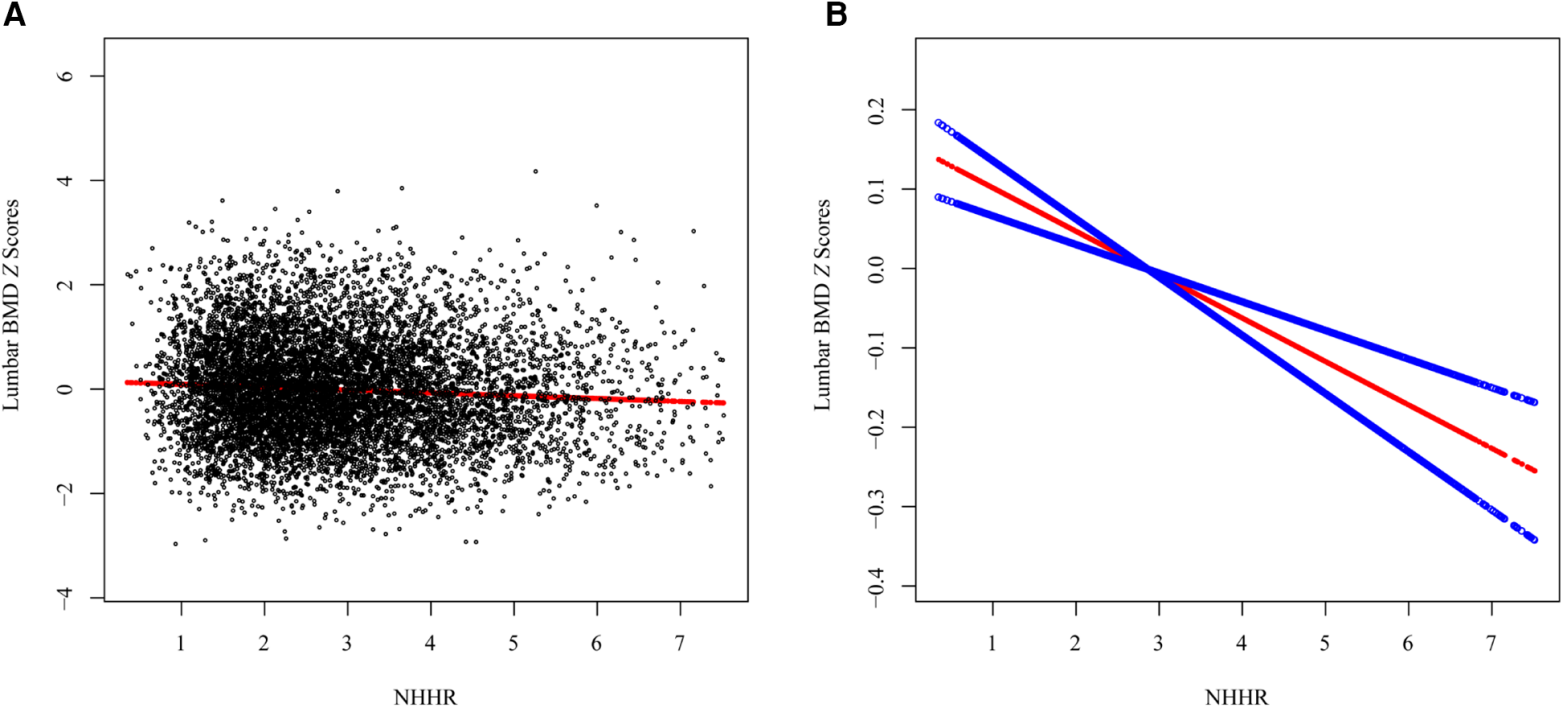
The association between the NHHR and lumbar spine BMD *Z* scores. (**A**) Each black point represents a sample, (**B**) Smooth curve fitting using GAM. The red line represents the smooth curve fit, and the blue line represents its 95% CI.

The association between NHHR and lumbar BMD *Z* scores was assessed by three multivariate linear regression models (Table 2). The association between NHHR and lumbar BMD *Z* scores was consistently and significantly negative across all models (Model 1: *β* = −0.039, 95% CI: −0.055, −0.023, *p* < 0.001; Model 2: *β* = −0.045, 95% CI: −0.062, −0.027, *p *< 0.001; Model 3: *β* = −0.042, 95% CI: −0.061, −0.023, *p* < 0.001). The trend remained statistical significance among the NHHR quartile groups (all *p* for trend <0.001), with participants in Q3 and Q4 having progressively lower lumbar BMD *Z* scores compared to those in Q1 (all *p* < 0.01). The smooth curve fits and GAM showed a linear association between NHHR and lumbar BMD *Z* scores based on the Model 3 (Figure 3).

**Table 2:** The association between NHHR and lumbar spine BMD *Z*-scores.

Exposure	Model 1, *β* (95% CI)	Model 2, *β* (95% CI)	Model 3, *β* (95% CI)
	*p*-value	*p*-value	*p*-value
NHHR	−0.039 (−0.055, −0.023) < 0.001	−0.045 (−0.062, −0.027) < 0.001	−0.042 (−0.061, −0.023) < 0.001
NHHR Quartile			
Q1 (0.36–1.90)	Reference	Reference	Reference
Q2 (1.91–2.65)	−0.046 (−0.105, 0.012) 0.121	−0.049 (−0.108, 0.010) 0.102	−0.054 (−0.114, 0.006) 0.076
Q3 (2.66–3.64)	−0.081 (−0.139, −0.023) 0.006	−0.094 (−0.153, −0.034) 0.002	−0.094 (−0.156, −0.033) 0.003
Q4 (3.65–7.53)	−0.147 (−0.206, −0.088) < 0.001	−0.165 (−0.228, −0.103) < 0.001	−0.156 (−0.223, −0.088) < 0.001
*p* for trend	<0.001	<0.001	<0.001

### 3.3 Subgroup analysis and interaction test

To determined whether the association between NHHR and lumbar BMD *Z* scores persists across specific populations, subgroups were stratified by age, sex, race, marital status, education level, BMI, waist circumference, smoking status, hypertension, DM status, V/MWA, and anti-hyperlipidemic drugs usage. A consistently negative association was observed across age, marital status, waist circumference, and V/MWA subgroups, suggesting that differences in covariates such as sex, race, education level, BMI, smoking status, hypertension, DM status, and the usage of anti-hyperlipidemic drugs may influence the linear relationship between NHHR and lumbar BMD *Z* scores (Supplementary Table S1). Interaction tests confirmed that the linear relationship between NHHR and lumbar BMD *Z* scores was significantly influenced by BMI (*p* for interaction = 0.012) and hypertension (*p* for interaction *= *0.047).

### 3.4 Non-linear relationships

**Figure 4:**
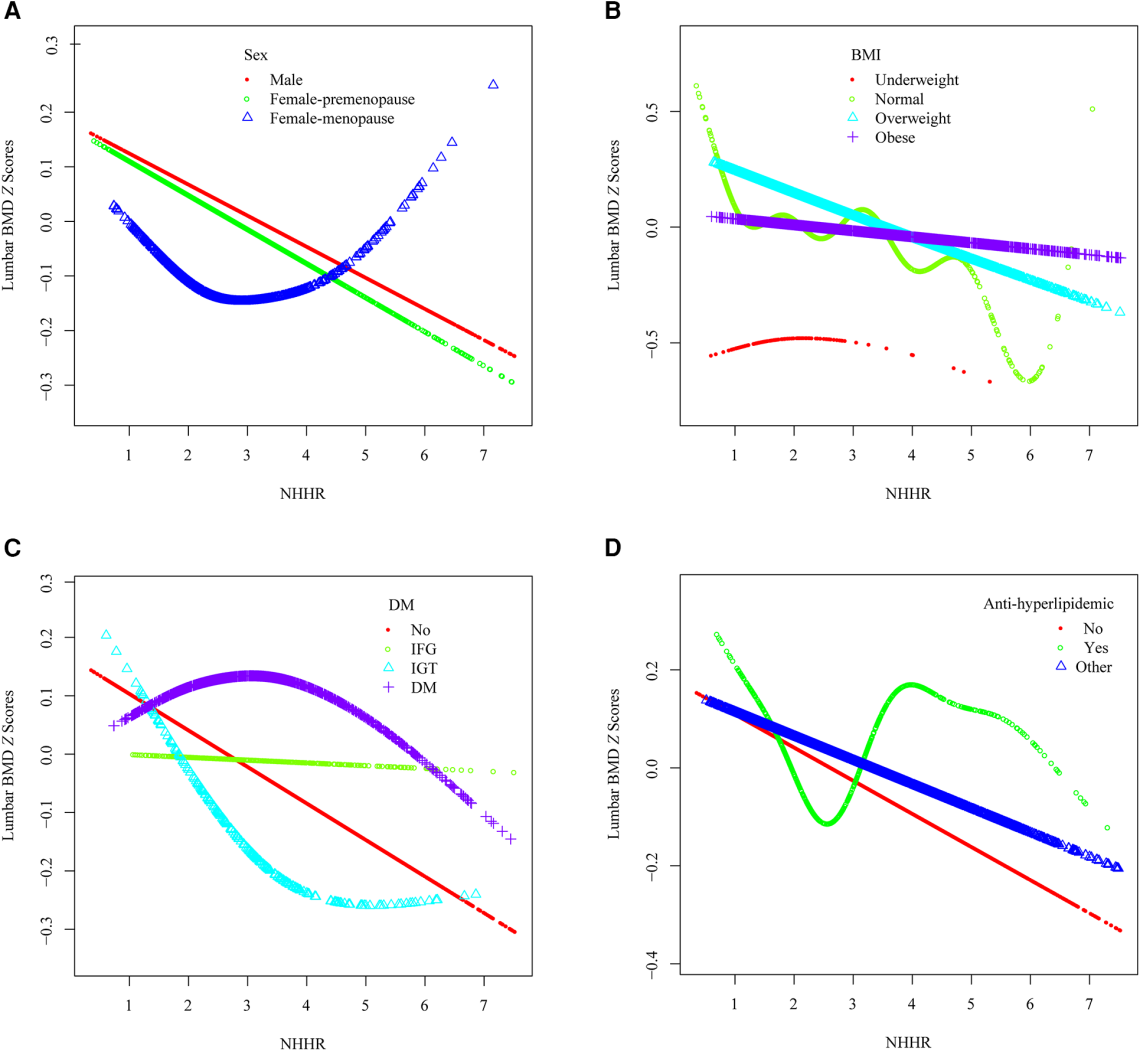
The association between the NHHR and lumbar spine BMD *Z* scores within specific populations. (**A**) Stratified by sex, (**B**) Stratified by BMI, (**C**) Stratified by DM, (**D**) Stratified by anti-hyperlipidemic drugs usage.

To detect the non-linear relationships of NHHR and lumbar BMD *Z* scores in the subgroups and further confirm the results, a GAM and smooth curve fitting based on the fully adjusted model (Model 3) were conducted. In addition to the significant interaction of BMI and hypertension, several potential demographic and comorbidity factors were explored (Figure 4).

A U-shaped association between NHHR and lumbar BMD *Z* scores in menopause females was identified (Figure 4A), with an inflection point at 4.891 (Table 3). Threshold effect analysis revealed a significant negative correlation before the inflection point (*β* = −0.096, 95% CI: −0.188, −0.005, *p =* 0.038) and a significant positive correlation afterward (*β* = 0.715, 95% CI: 0.245, 1.185, *p =* 0.003).

**Table 3:** Threshold effect analysis of NHHR on lumbar spine BMD *Z* scores within specific populations.

NHHR	*β* (95%CI) *p* value				
	Female-menopause	Underweight	IGT	DM	Anti-hyperlipidemic drugs taken
Fitting by the standard linear model					
	−0.020 (−0.098, 0.058) 0.616	−0.091 (−0.316, 0.135) 0.432	−0.073 (−0.187, 0.040) 0.207	−0.022 (−0.072, 0.028) 0.395	0.034 (−0.035, 0.103) 0.333
Fitting by the two-piecewise linear model					
Inflection point (K)	4.891	1.4	3.163	1.6	2.16
NHHR < K	−0.096 (−0.188, −0.005) 0.038	1.899 (0.597, 3.201) 0.005	−0.311 (−0.560, −0.063) 0.015	0.915 (0.207, 1.623) 0.012	−0.321 (−0.636, −0.006) 0.046
NHHR > K	0.715 (0.245, 1.185) 0.003	−0.256 (−0.498, −0.014) 0.041	0.081 (−0.102, 0.263) 0.387	−0.046 (−0.099, 0.007) 0.092	0.089 (0.006, 0.173) 0.037
Log likelihood ratio	0.002	<0.001	0.027	0.009	0.021

A reverse U-shaped association between NHHR and lumbar BMD *Z* scores was found in underweight participants (Figure 4B), with an inflection point at 1.4 (Table 3). Threshold effect analysis revealed a significant positive correlation before the inflection point (*β* = 1.899, 95% CI: 0.597, 3.201, *p =* 0.005), and a significant negative correlation afterward (*β* = −0.256, 95% CI: −0.498, −0.014, *p =* 0.041).

An L-shaped association between NHHR and lumbar BMD *Z* scores was detected in participants with IGT (Figure 4C), with an inflection point at 3.163 (Table 3). A significant negative correlation was present before the inflection point (*β* = −0.311, 95% CI: −0.560, −0.063, *p =* 0.015), while no significant correlation was found after the inflection point (*β* = 0.081, 95% CI: −0.102, 0.263, *p =* 0.387).

A reverse L-shaped relationship was observed between NHHR and lumbar BMD *Z* scores in participants with DM (Figure 4C), with an inflection point at 1.6 (Table 3). A significant positive correlation was observed before the inflection point (*β* = 0.915, 95% CI: 0.207, 1.623, *p =* 0.012), with no significant correlation afterward (*β* = −0.046, 95% CI: −0.099, 0.007, *p =* 0.092).

Among participants using anti-hyperlipidemic drugs, a U-shaped association between NHHR and lumbar BMD *Z* scores was observed (Figure 4D, Table 3). Before the inflection point at 2.16, a negative correlation was evident (*β* = −0.321, 95% CI: −0.636, −0.006, *p =* 0.046), followed by a significant positive correlation after the inflection point (*β* = 0.089, 95% CI: 0.006, 0.173, *p =* 0.037).

## 4 Discussion

To our knowledge, this is the first study to investigate the association between arteriosclerosis index and lumbar BMD in a population-based setting. Despite initially statistically significant negative associations were observed between NHHR and lumbar BMD *Z* scores in the multivariate linear regression models, these associations varied within specific populations identified through subgroup analyses.

It is important to note that a non-linear and U-shaped association was seen among menopause females. In this population, an NHHR value greater than 4.891 appeared to be a protective factor against bone mass loss. Menopause is linked to dyslipidemia and an elevated risk of cardiovascular disease and osteoporosis due to a deterioration in lipid profile during the transition to postmenopausal status, characterized by increases in TC, LDL-C and TG along with a net reduction in HDL-C (30). A multi-center study reported that a high HDL-C level is an independent risk factor for bone loss in both males and females (31). However, other studies have demonstrated a positive correlation between HDL-C and lumbar BMD in females (11) or postmenopausal females (8). In addition, the association between LDL-C and BMD among postmenopausal females remains uncertain (8, 32, 33). Explanations for the above controversial results may be attributed to differences in participants and assessment of BMD. To our knowledge, there is no evidence of a U-shaped relationship between TC and BMD or between HDL-C and BMD. This study identified the optimal inflection point of NHHR in specific populations, providing a beneficial range for regulating lipid levels. NHHR was regarded as a novel instrument for elucidating the relationship between lipids and lumbar BMD, emphasizing the importance of other atherogenic cholesterols in bone metabolism.

Notably, the detrimental effect of NHHR on lumbar BMD is most pronounced among the overweight population. Conversely, NHHR exhibits a positive association with lumbar BMD among the underweight cohort when NHHR values are below 1.4. Obesity can have significant consequences on various organs and systems, with its effects on bone being particularly controversial (34). Lower BMI has been associated with an increased risk of osteoporosis, and higher body weight is believed to provide protection against fractures (35). However, obesity and its comorbidities such as dyslipidemia, type 2 diabetes, and metabolic syndrome, may contribute to affect bone health (36). Elevated NHHR within eutrophic ranges poses a greater risk for bone loss; however, this effect diminishes with progression toward morbid obesity, where other comorbidities exert a more substantial influence on BMD.

The negative association between NHHR and lumbar BMD was more likely to be seen among the population without overt health issues (hypertension, IFG, IGT, or DM). Hypertension has been identified as being associated with decreased BMD (37). In addition, prolonged usage of non-thiazide diuretics among hypertensive individuals might contribute to diminished BMD due to increased urinary calcium excretion, which decreases calcium availability for bone formation (38). Conversely, thiazide diuretics reduce renal calcium excretion by promoting calcium reabsorption in the distal convoluted tubules, reduce bone turnover by lowering parathyroid hormone levels, and may stimulate osteoblast differentiation while inhibiting osteoclast formation (38, 39). The correlation between DM and bone health is intricate. Most studies suggest normal or superior trabecular bone structure in patients with DM, although some have reported a decreased lumbar spine trabecular bone score and an increased risk of spine fracture (40). Importantly, both hypertension and DM are acknowledged risk factors for cardiovascular disease, exerting direct or indirect influences on lipid metabolism. Therefore, the relationship between NHHR and lumbar BMD is further complicated by the interference of diabetes or hypertension.

Lipid-lowering therapy, such as commonly prescribed statin medications, undeniably influences the linear association between NHHR and lumbar BMD. Preclinical studies have demonstrated that statins mitigate bone loss by inhibiting osteoclastogenesis and promoting osteoblast development, potentially exerting an additional favorable impact on BMD (41, 42). Statins upregulate the expression of key mediators in bone metabolism, including ALP, bone morphogenetic protein-2, transforming growth factor-beta, type I collagen, collagenase-1, and glucocorticoids (42). Upon excluding individuals taking glucocorticoids and thyroid hormones, no significant influence of medications other than lipid-lowering drugs was found on the relationship between NHHR and lumbar BMD.

This study possesses several notable strengths. Firstly, utilizing *Z* scores provides greater precision in predicting fracture risk, benefiting from a standardized reference across age, gender, and race (43). Second, two critical time-frames of bone loss were examined: 35 years old, marking the onset of bone aging, and menopause, when bone loss accelerates. Third, the liner association between NHHR and lumbar BMD was observed in an ostensibly healthy population, enhancing the relevance of the findings to the general population.

However, several limitations must be acknowledged. First, the cross-sectional design of the NHANES dataset precludes establishing causality between NHHR and lumbar BMD. Second, BMD scans in the NHANES 2011–2018 were conducted on adults up to age 59 years, whereas routine BMD measurements are clinically recommended around age 65 years, potentially leading to a failure to capture the period of highest risk for osteoporosis (44). Third, serum estradiol data from the NHANES 2011-2012 and NHANES 2017–2018 were unavailable. Fourth, despite adjustments for numerous confounding variables, the influence of unmeasured or unknown confounders on the results cannot be entirely ruled out such as diet quality, nutritional supplements, exercise types. Finally, our study focuses solely on lumbar BMD and does not consider other sites like the thoracic spine, hip, or forearm. We will conduct further studies to provide a more comprehensive understanding of this relationship.

## 5 Conclusion

This study revealed a negative association between NHHR and lumbar BMD. In population with specific condition or statuses, NHHR should be tailored to individual levels to mitigate bone loss. Individuals at heightened risk of cardiovascular disease are encouraged to prioritize their bone health. This study introduces a novel tool for monitoring lipid levels and mitigating bone loss, with notable clinical implications.

## Data Availability

Publicly available datasets were analyzed in this study. This data can be found here: https://www.cdc.gov/nchs/nhanes/.
