# Supplementary material for: Association between arteriosclerosis index and lumbar bone mineral density in U.S adults: a cross-sectional study from the NHANES 2011–2018
Source: Front Cardiovasc Med. 2024 Aug 1;11:1459062. doi: 10.3389/fcvm.2024.1459062 (PMC11324549; doi:10.3389/fcvm.2024.1459062)
Supplement: Supplementary file 1 [file Table1.docx]

| **Table S1** Subgroup analysis and interaction test | | | | | | | |
| --- | --- | --- | --- | --- | --- | --- | --- |
| **Variable** | ***β* (95% CI)** | | | | ***p* for trend** (character2integer) | ***p* for trend** (Median value) | ***p* for interaction** |
|  | **NHHR Quartile** | | | |  |  |  |
|  | **Q1** | **Q2** | **Q3** | **Q4** |  |  |  |
| Age-stratified |  |  |  |  |  |  | 0.867 |
| 20 to 35 years old | Reference | -0.066(-0.196,0.064) | -0.129(-0.260,0.003) | -0.159(-0.318,0.000) | 0.028 | 0.032 |  |
| 36 to 59 years old | Reference | -0.059(-0.186, 0.068) | -0.093(-0.201, 0.015) | -0.174(-0.283,-0.065) | 0.002 | 0.002 |  |
| Sex |  |  |  |  |  |  | 0.057 |
| Male | Reference | 0.097(-0.034, 0.227) | -0.043(-0.149, 0.063) | -0.121(-0.226,-0.017) | <0.001 | <0.001 |  |
| Female-premenopause | Reference | -0.146(-0.289,-0.003) | -0.11(-0.218,-0.002) | -0.166(-0.318,-0.014) | 0.015 | 0.021 |  |
| Female-menopause | Reference | -0.275(-0.568, 0.018) | -0.143(-0.501, 0.214) | -0.168(-0.563, 0.227) | 0.582 | 0.663 |  |
| Race/ethnicity |  |  |  |  |  |  | 0.83 |
| Non-Hispanic White | Reference | -0.042(-0.176, 0.092) | -0.107(-0.207,-0.007) | -0.133(-0.256,-0.010) | 0.012 | 0.02 |  |
| Non-Hispanic Black | Reference | -0.029(-0.150, 0.092) | -0.076(-0.194, 0.042) | -0.148(-0.320, 0.024) | 0.073 | 0.078 |  |
| Mexican American | Reference | -0.119(-0.313, 0.076) | -0.024(-0.210, 0.162) | -0.158(-0.395, 0.079) | 0.314 | 0.267 |  |
| Other | Reference | -0.138(-0.279, 0.004) | -0.204(-0.340,-0.068) | -0.34(-0.489,-0.192) | <0.001 | <0.001 |  |
| Married |  |  |  |  |  |  | 0.363 |
| No | Reference | -0.054(-0.167, 0.060) | -0.029(-0.131, 0.072) | -0.164(-0.302,-0.025) | 0.042 | 0.033 |  |
| Yes | Reference | -0.064(-0.199, 0.071) | -0.183(-0.306,-0.060) | -0.19(-0.332,-0.048) | 0.003 | 0.006 |  |
| Education |  |  |  |  |  |  | 0.522 |
| < High school | Reference | 0.017(-0.181, 0.215) | -0.088(-0.251, 0.074) | -0.041(-0.239, 0.158) | 0.513 | 0.608 |  |
| High school | Reference | 0.013(-0.147, 0.174) | -0.061(-0.232, 0.110) | -0.225(-0.409,-0.042) | 0.009 | 0.006 |  |
| > High school | Reference | -0.081(-0.201, 0.038) | -0.11(-0.210,-0.009) | -0.171(-0.289,-0.054) | 0.005 | 0.006 |  |
| BMI |  |  |  |  |  |  | 0.012 |
| Normal | Reference | 0.004(-0.144, 0.152) | 0.031(-0.105, 0.168) | -0.221(-0.432,-0.010) | 0.178 | 0.097 |  |
| Overweight | Reference | -0.152(-0.288,-0.017) | -0.228(-0.384,-0.072) | -0.34(-0.488,-0.192) | <0.001 | <0.001 |  |
| Obese | Reference | 0.006(-0.172, 0.184) | -0.034(-0.194, 0.126) | -0.011(-0.182, 0.160) | 0.811 | 0.868 |  |
| Underweight | Reference | -0.414(-0.835, 0.006) | 0.34(-0.205, 0.884) | -0.664(-1.387, 0.059) | 0.164 | 0.148 |  |
| Waistline |  |  |  |  |  |  | 0.411 |
| Normal | Reference | -0.043(-0.164, 0.077) | -0.092(-0.207, 0.024) | -0.226(-0.365,-0.087) | 0.002 | 0.001 |  |
| Central obesity | Reference | -0.063(-0.220, 0.094) | -0.106(-0.232, 0.019) | -0.126(-0.261, 0.008) | 0.049 | 0.065 |  |
| Smoke |  |  |  |  |  |  | 0.736 |
| Never | Reference | -0.062(-0.167, 0.042) | -0.088(-0.178, 0.002) | -0.114(-0.234, 0.007) | 0.044 | 0.068 |  |
| Former | Reference | -0.053(-0.309, 0.203) | -0.155(-0.374, 0.063) | -0.339(-0.576,-0.103) | 0.002 | 0.001 |  |
| Now | Reference | -0.081(-0.252, 0.089) | -0.142(-0.332, 0.047) | -0.167(-0.339, 0.005) | 0.065 | 0.075 |  |
| Hypertension |  |  |  |  |  |  | 0.047 |
| No | Reference | -0.07(-0.177, 0.036) | -0.105(-0.191,-0.019) | -0.225(-0.345,-0.106) | <0.001 | <0.001 |  |
| Yes | Reference | -0.023(-0.163, 0.118) | -0.091(-0.241, 0.059) | -0.027(-0.183, 0.128) | 0.675 | 0.844 |  |
| DM |  |  |  |  |  |  | 0.234 |
| No | Reference | -0.064(-0.166, 0.037) | -0.092(-0.170,-0.013) | -0.198(-0.303,-0.093) | <0.001 | <0.001 |  |
| IFG | Reference | -0.225(-0.606, 0.157) | -0.461(-0.729,-0.193) | -0.203(-0.550, 0.143) | 0.544 | 0.811 |  |
| IGT | Reference | -0.126(-0.845, 0.594) | -0.364(-0.928, 0.201) | -0.058(-0.807, 0.691) | 0.877 | 0.921 |  |
| DM | Reference | 0.151(-0.105,0.407) | 0.034(-0.230,0.297) | 0.023(-0.252,0.298) | 0.754 | 0.731 |  |
| Activity |  |  |  |  |  |  | 0.595 |
| No | Reference | -0.055(-0.181, 0.071) | -0.081(-0.175, 0.012) | -0.202(-0.338,-0.065) | 0.003 | 0.003 |  |
| Yes | Reference | -0.071(-0.196, 0.055) | -0.136(-0.242,-0.029) | -0.159(-0.276,-0.043) | 0.005 | 0.011 |  |
| Anti-hyperlipidemic drugs |  |  |  |  |  |  | 0.136 |
| No | Reference | -0.043(-0.158, 0.072) | -0.077(-0.159, 0.005) | -0.17(-0.278,-0.063) | 0.001 | 0.001 |  |
| Yes | Reference | -0.128(-0.395, 0.140) | -0.04(-0.318, 0.239) | 0.113(-0.215, 0.442) | 0.382 | 0.297 |  |
| Other | Reference | -0.086(-0.237, 0.064) | -0.176(-0.321,-0.030) | -0.227(-0.378,-0.076) | 0.002 | 0.003 |  |
